# Hormone-related risk factors for breast cancer in women under age 50 years by estrogen and progesterone receptor status: results from a case–control and a case–case comparison

**DOI:** 10.1186/bcr1514

**Published:** 2006-07-17

**Authors:** Huiyan Ma, Leslie Bernstein, Ronald K Ross, Giske Ursin

**Affiliations:** 1 Department of Preventive Medicine, Keck School of Medicine, University of Southern California, 1441 Eastlake Avenue, USC/Norris Comprehensive Cancer Center, Los Angeles, California 90089-9175, USA; 2 Department of Nutrition, University of Oslo, Norway

## Abstract

**Introduction:**

It has been suggested that hormonal risk factors act predominantly on estrogen receptor and progesterone receptor (ER/PR)-positive breast cancers. However, the data have been inconsistent, especially in younger women.

**Methods:**

We evaluated the impact of age at menarche, pregnancy history, duration of breastfeeding, body mass index, combined oral contraceptive use, and alcohol consumption on breast cancer risk by ER/PR status in 1,725 population-based case patients and 440 control subjects aged 20 to 49 years identified within neighborhoods of case patients. We used multivariable unconditional logistic regression methods to conduct case–control comparisons overall as well as by ER/PR status of the cases, and to compare ER^+^PR^+ ^with ER^-^PR^- ^case patients.

**Results:**

The number of full-term pregnancies was inversely associated with the risk of ER^+^PR^+ ^breast cancer (*p*_trend _= 0.005), whereas recent average alcohol consumption was associated with an increased risk of ER^+^PR^+ ^breast cancer (*p*_trend _= 0.03). Neither of these two factors was associated with the risk of ER^- ^PR^- ^breast cancer. Late age at menarche and a longer duration of breastfeeding were both associated with decreased breast cancer risk, irrespective of receptor status (all *p*_trend_≤ 0.03).

**Conclusion:**

Our results suggest that the number of full-term pregnancies and recent alcohol consumption affect breast cancer risk in younger women predominantly through estrogen and progesterone mediated by their respective receptors. Late age at menarche and breastfeeding may act through different hormonal mechanisms.

## Introduction

It has been well documented that estrogen and progesterone are important in breast tumorigenesis [1-3], and their effects on the breast are mediated by their respective receptors, the estrogen receptor (ER) and the progesterone receptor (PR) [4-7]. Furthermore, it has been hypothesized that hormone-related risk factors that reflect exposure to estrogen and progesterone may be predominantly associated with breast tumors that express ER and PR, but not with those lacking ER and PR expression [8-14]. Several epidemiological studies have examined this hypothesis by ER and PR status separately or jointly [15-17], and a review from 2004 [17] concluded that early age at menarche, nulliparity, and delayed childbearing were associated with an increased risk for receptor-positive breast cancer, but not with receptor-negative breast cancer. However, in the prospective data from the Nurses' Health Study [18], the adverse effect of nulliparity was confined to ER^+^PR^+ ^breast cancer, but early age at menarche was associated with an increased risk of both ER^+^PR^+ ^and ER^- ^PR^- ^breast cancer and the adverse effect of delayed childbearing was observed for ER^- ^PR^- ^but not ER^+^PR^+ ^breast cancer. Results from studies of young women under the age of 50 years [19-21] or premenopausal women [12,22] are even less consistent. To help shed light on the issue, we evaluated hormone-related risk factors for breast cancer by receptor subtypes (ER^+^PR^+ ^and ER^- ^PR^-^) in a large study of women aged 20 to 49 years, using both case–control and case–case comparisons.

## Materials and methods

### Case patients

Case patients were identified through the Los Angeles County Cancer Surveillance Program (CSP), the population-based cancer registry that is part of the National Cancer Institute's Surveillance, Epidemiology, and End Results (SEER) cancer registry program. Eligible case patients were US-born English-speaking, white (including Hispanic) or African-American, female residents of Los Angeles County aged 20 to 49 years when diagnosed with histologically confirmed first primary invasive breast cancer. We identified 2,882 eligible case patients (2,534 white and 348 African-American). White patients were diagnosed between February 1998 and May 2003 and African-American patients were diagnosed between January 2000 and May 2003. We were unable to interview 1,088 of the 2,882 eligible case patients (38%) because of patient refusal (*n *= 428), no longer living in Los Angeles County (*n *= 37), inability to be located (*n *= 88), death (*n *= 38), serious illness or disability (*n *= 18), physician refusal (*n *= 50), or inability to schedule the interview within 18 months of diagnosis (*n *= 429). We successfully interviewed 1,794 (62%) eligible case patients (1,585 white, 209 African-American).

### Control subjects

Because this study was originally designed as a case–case study to examine genetic risk factors for breast cancer, we did not collect control subjects for all case patients. The control subjects who were recruited were matched by race and age (within 5 years and aged 20 to 49 years) to a subset of case patients who were diagnosed between July 2000 and March 2003. Control subjects were US-born English-speaking white or African-American women who had never been diagnosed with invasive or *in situ *breast cancer. They were identified by using a neighborhood walk algorithm that we had used in previous case–control studies [23,24]. Field staff conducted walks according to a predefined pattern in the neighborhoods where case patients lived at the time of their diagnoses. The houses on the immediate blocks surrounding the home of the case patients were excluded from the walk. Residences were visited sequentially and information on potentially eligible women was obtained. If no one was home at the time of the visit, we left a request for information at the door, and we sought further information from neighbors so that we could contact the residents later. If we received no response to our written request, we sent additional letters until we were able to determine whether an eligible woman lived at the address in question. Detailed records were maintained to determine the number of housing units contacted in order to identify and interview a control subject. By the end of the study we had identified 603 eligible control subjects for the 1,108 case patients (1,018 white, 90 African-American). We were unable to interview 159 of the 603 control subjects (26%) as a result of subject refusal (*n *= 77), no longer living in Los Angeles County (*n *= 18), death (*n *= 1), serious illness (*n *= 2), or inability to schedule the interview within 18 months from the date of initial household contact (*n *= 61). We successfully interviewed 444 (74%) of eligible control subjects (409 white, 35 African-American). On average, 20 houses were canvassed to find an eligible control subject who agreed to be interviewed.

### Data collection

All participants were interviewed in person with the use of a structured questionnaire, which was a modified version of the questionnaire used for the Women's Contraceptive and Reproductive Experiences (CARE) Study [25]. Our questionnaire included reproductive history (including breastfeeding), detailed histories of oral contraceptive use and alcohol consumption, family breast cancer history, demographics, and other factors. Information was recorded up to a predetermined reference date for each participant. The reference date was the date of diagnosis for case patients and the date of initial household contact for control subjects. All participants signed informed consent documents before interview. The study protocol was approved by the federally approved Institutional Review Board at the University of Southern California Medical Center.

Information on ER and PR status for interviewed case patients was obtained from the CSP. Among the 1,794 case patients, 1,510 (84%) had information on both ER and PR status; 91 of these were reported as weakly or borderline positive (84 cases) or undecided (7 cases) for either ER or PR. Among the other 1,419 case patients, 881 (62%) were ER^+^PR^+^, 92 (6%) were ER^+^PR^-^, 41 (3%) were ER^- ^PR^+^, and 405 (29%) were ER^- ^PR^- ^.

### Data analyses

We compared demographic and hormone-related risk factors among case patients with known ER and PR information, borderline positive or undecided results, and patients without ER or PR information, with the use of *F *tests for differences in means and Pearson χ^2 ^tests for differences in frequency distributions. When the two-sided *p *value comparing all three groups was less than 0.05, we also performed pairwise comparisons by using Bonferroni *t *tests or Pearson χ^2 ^tests, imposing a Bonferroni correction to the *p *value, restricting the overall type I error to 5% by setting as statistically significant only two-sided *p *values less than 0.017 for each pairwise comparison [26].

Analyses were conducted to assess the association between breast cancer and the following factors: age at menarche, number of full-term (greater than 26-week gestation) pregnancies, age at first full-term pregnancy (defined for each woman as the age at which that pregnancy ended), duration of breastfeeding, body mass index (BMI, kg/m^2^) one year before the participant's reference date, duration of combined oral contraceptive (COC) use, alcohol drinking status during reference age (never, former, and current – that is, drinking alcohol during reference age), and the average number of alcoholic drinks per week in the 5-year period that ended 2 years before the reference age. One alcoholic drink was defined as 12 ounces (355 ml) of beer, 4 ounces (118 ml) of wine, or 1.5 ounces (44 ml) of liquor.

We conducted case–control comparisons for overall, ER^+^PR^+^, and ER^- ^PR^- ^case patients with control subjects, and also compared ER^+^PR^+ ^with ER^- ^PR^- ^case patients. We used polytomous logistic regression [27] to compare ER^+^PR^+ ^and ER^- ^PR^- ^case patients simultaneously with control subjects. We used a multivariable unconditional logistic regression approach [27] for the comparisons of all case patients with controls, and ER^+^PR^+ ^with ER^- ^PR^- ^case patients. We estimated multivariable adjusted odds ratios (ORs) and 95% confidence intervals (CIs). Tests for trend were conducted by fitting ordinal values corresponding to categories of exposure in our models and testing whether the coefficient (slope of the dose response) differed from zero.

Adjustment was made for race (white, African-American), age (less than 30, 30 to 34, 35 to 39, 40 to 44, 45 to 49 years), and education (high school or lower, technical school or some college, college graduate) in all our models. Additionally, multivariable models included variables selected a priori as potential confounders: first-degree family history of breast cancer (no first-degree family history, mother or sister with breast cancer, unknown first-degree family history), age at menarche (11 or less, 12, 13, at least 14 years), gravidity (never pregnant, ever pregnant), number of full-term pregnancies (never full-term pregnant, one, two, three, at least four full-term pregnancies), BMI 1 year before reference date (less than 25, 25 to 29, 30 to 34, at least 35 kg/m^2^), COC use (never, less than 1, 1 to 4, 5 to 9, at least 10 years), the average number of alcoholic drinks per week in the recent 5 years (never, less than 3, 3 to 5, 6 to 11, at least 12 drinks, drinking alcohol but not within the 5 years of interest), and a three-category variable combining menopausal status and hormone therapy use (premenopausal or – among postmenopausal women – never used hormone therapy, or had used estrogen therapy or estrogen plus progestin therapy). When estimating the effects of parity or restricting analyses to parous women, we did not include gravidity in our models. A single model was fitted to assess the joint effects of age at first full-term pregnancy (less than 22, 22 to 27, 28 to 31, at least 32 years) and breastfeeding duration (0, less than 1, 1 to 6, 7 to 23, at least 24 months) among parous women. All variables were included as categorical variables in the models. In reporting the results of trend tests, we considered a two-sided *p *value of less than 0.05 as statistically significant. All analyses were performed with the SAS statistical package (Version 9.0, SAS Institute, Cary, NC, USA).

To maintain a constant sample size for all analyses, we excluded 69 case patients and 4 control subjects for the following reasons: missing information on educational attainment (15 cases and 1 control), age at menarche (3 cases), parity (4 cases and 1 control), duration of breastfeeding (6 cases), BMI (4 cases), duration of COC use (17 cases and 1 control), recent alcohol consumption (14 cases); missing information on two or more of these factors (6 cases and 1 control). This resulted in 1,725 case patients and 440 control subjects available for the overall case–control analyses. Among the 1,725 case patients, 1,449 (84%) had information on both ER and PR status; 83 of these were reported as weakly, borderline positive or undecided for either ER or PR and were excluded from the analyses by receptor subtypes. Among the other 1,366 case patients, 63% were ER^+^PR^+^, 6% were ER^+^PR^-^, 3% were ER^-^PR^+^, and 28% were ER^- ^PR^- ^. The frequency distribution across receptor subtypes was similar to those reported by previous studies conducted within the SEER registries [21,28]. In addition, our numbers are very similar to those that we observed among the white Los Angeles cases in the Women's CARE Study. This may be the most appropriate comparison, because 88% of the cases in the present study were white. We excluded ER^+^PR^- ^and ER^- ^PR^+ ^subtypes from the analyses by receptor status because they were rare. There were therefore 1,239 remaining for the analyses by ER and PR status (854 ER^+^PR^+ ^and 385 ER^- ^PR^-^).

As described above, our control subjects were identified through matching to a subset of the cases. An alternative to conditional logistic regression in matched studies with disproportionate numbers of cases and controls is to break the match and conduct unconditional logistic regression with detailed adjustment for the matching factors [29]. We conducted both detailed stratified analyses, with strata defined by race, age categories (five-year categories) and education as a proxy of socioeconomic status/neighborhood as well as standard unconditional logistic regression with adjustment for the same factors. Because the results remained the same, we chose to use multivariable unconditional logistic regression for all our analyses. We also repeated all the analyses with the subset of the cases used to identify the controls. Again, the results were essentially identical with the overall analyses; we therefore present results based on all case patients.

## Results

Table 1 shows the distributions of demographic and hormone-related risk factors among case patients stratified by availability of ER/PR information and among control subjects. Age at breast cancer diagnosis (*p*_*F *test _= 0.0001), education level ( = 0.01), age at first full-term pregnancy (*p*_*F *test _= 0.002), and percentage of case patients who ever breastfed ( = 0.01) differed across the three case groups. Pairwise analyses revealed that the differences were between case patients with known ER/PR status and those without ER/PR information. In comparison with case patients without ER/PR information, case patients with known ER/PR status were on average 1.5 years younger at diagnosis (*p*_*t *test _*<*0.0001), better educated ( = 0.001), on average 1.8 years older at first full-term pregnancy (*p*_*t *test _= 0.001), and had a higher percentage of case patients who had breastfed ( = 0.004).

### Age at menarche

Age at menarche was negatively associated with breast cancer risk regardless of ER/PR status (all *p*_trend_≤ 0.008; Table 2). In comparison with women who had menarche before the age of 12 years, later age at menarche (at least 14 years) was associated with an approximately 40% reduced risk of breast cancer among the ER^+^PR^+ ^case patients, ER^- ^PR^- ^case patients, and all case patients combined. The associations with age at menarche did not differ between ER^+^PR^+ ^and ER^- ^PR^- ^case patients (*p*_trend _= 0.85).

### Parity

A protective effect of parity was confined to women with ER^+^PR^+ ^breast cancer (Table 2). The OR of ER^+^PR^+ ^breast cancer decreased with increasing number of full-term pregnancies (*p*_trend _= 0.005). Parous women who had four or more full-term pregnancies had an approximately 50% reduction in the risk of ER^+^PR^+ ^breast cancer compared with women who never had a full-term pregnancy. ER^+^PR^+ ^case patients were less likely to have had many full-term pregnancies than ER^- ^PR^- ^case patients (*p*_trend _= 0.09).

### Age at first full-term pregnancy

A slight increase in risk for ER^+^PR^+ ^breast cancer and a reduced risk for ER^- ^PR^- ^breast cancer was observed with increasing age at first full-term pregnancy, but none of the confidence limits for the risk estimates excluded 1.0 and no linear trend in risk was observed for either cancer type (ER^+^PR^+^, *p*_trend _= 0.49; ER^- ^PR^-^, *p*_trend _= 0.08; Table 2). ER^+^PR^+ ^case patients were more likely to have had a late first full-term pregnancy than ER^-^PR^- ^case patients (*p*_trend _= 0.009).

### Breastfeeding

Duration of breastfeeding was negatively associated with breast cancer risk regardless of ER/PR status (all *p*_trend_≤ 0.03; Table 2). In the case–case analysis, duration of breastfeeding was not associated with ER/PR status (*p*_trend _= 0.63).

### Body mass index 1 year before reference date

Increasing BMI was associated with a non-statistically significant decreasing risk of ER^+^PR^+ ^breast cancer (*p*_trend _= 0.20) but was not associated with ER^- ^PR^- ^breast cancer (Table 2). Moreover, among premenopausal women, increasing BMI was marginally statistically significantly associated with a decreasing risk of ER^+^PR^+ ^breast cancer (*p*_trend _= 0.08) but not ER^-^PR^- ^breast cancer (*p*_trend _= 0.54). In comparison with premenopausal women who had a low BMI (less than 25 kg/m^2^), the OR among premenopausal women in the highest BMI category (at least 35 kg/m^2^) was 0.58 (95% CI 0.34 to 1.00) for ER^+^PR^+ ^breast cancer and 1.07 (95% CI 0.58 to 1.97) for ER^- ^PR^- ^breast cancer. ER^+^PR^+ ^case patients were less likely to have had a higher BMI than ER^- ^PR^- ^case patients (*p*_trend _= 0.005).

### Combined oral contraceptive use

COC use was not associated with risk of either ER^+^PR^+ ^or ER^-^PR^- ^breast cancer (Table 2). Women who had used COC for 10 years or longer had a slightly higher OR of ER^- ^PR^- ^breast cancer (OR 1.27, 95% CI 0.75 to 2.14) but a lower OR of ER^+^PR^+ ^breast cancer (OR 0.76, 95% CI 0.49 to 1.18) compared with never users. ER^+^PR^+ ^case patients were less likely to have had longer duration of COC use than ER^- ^PR^- ^case patients (*p*_trend _= 0.008).

### Alcohol consumption

Alcohol drinking status during reference age was not associated with breast cancer risk (Table 2). However, the average number of alcoholic drinks per week in the recent 5 years was positively associated with ER^+^PR^+ ^breast cancer (*p*_trend _= 0.03), weakly associated with all types of cancer together (*p*_trend _= 0.12), and not associated with ER^- ^PR^- ^breast cancer (*p*_trend _= 0.42). Overall, ER^+^PR^+ ^case patients seemed more likely to have drunk larger quantities of alcohol than ER^- ^PR^- ^case patients, but the difference was not statistically significant (*p*_trend _= 0.23).

## Discussion

Overall in this study of women under age 50 years, we found associations that differed by ER/PR status for the number of full-term pregnancies, recent alcohol consumption, and possibly age at first full-term pregnancy and BMI. Risk reductions associated with late age at menarche and long duration of breastfeeding did not differ by ER/PR status. COC use was not associated with breast cancer risk in this study.

The magnitude of the protective effect from multiple full-term pregnancies for ER^+^PR^+ ^breast cancer in our study is similar to that observed in a previous study of young women [21], smaller than that observed in a study of premenopausal women (OR 0.44, 95% CI 0.26 to 0.75 of ER^+^PR^+ ^cancer for women having three or more live births compared with nulliparous women) [22], and greater than that in two studies of women under 45 years of age that did not find any associations with either receptor subtype [19,20]. A greater number of full-term pregnancies may protect against receptor-positive breast cancer by causing the full differentiation of breast epithelium [30], thereby reducing cyclical morphological change in breast tissue during the menstrual cycles [31].

Our results on age at first full-term pregnancy are consistent with the bulk of the evidence that early age at first full-term pregnancy is not a strong protective factor in young women. This may be due to a transient increase in breast cancer risk after pregnancy [32]. However, if early age at first full-term pregnancy protects against any subtype of breast cancer, our results suggest that it would be the receptor-positive cancer.

Five previous studies have examined the effect of BMI according to ER/PR status among young [19,20] or premenopausal [12,22,33] women. Two found that, when compared with the lowest category of BMI, the highest category of BMI was associated with a 23 to 29% reduction in risk of ER^+^PR^+ ^breast cancer but not ER^- ^PR^- ^breast cancer [19,22]. The other three studies found no statistically significant association with any receptor subtype [12,20,33]. Our data showed a 31% reduced risk of ER^+^PR^+ ^breast cancer, which did not quite reach statistical significance. A possible mechanism that would explain a protective effect of BMI on receptor-positive tumors is that high BMI results in anovulatory menstrual cycles with reduced exposure to ovarian hormones [34,35].

Among five previous epidemiological studies that examined the effect of alcohol consumption according to ER/PR status among young women [19,36] or premenopausal women [12,22,37], two found a 10 to 38% increase in risk of ER^+^PR^+ ^cancer, which was not statistically significant, for women at the highest category of alcohol consumption versus women who had never drunk alcohol [22,37]; one found an increase in risk of the ER^+^PR^- ^subtype [36]; and another found a non-statistically significant increase in risk of both ER^+^PR^+ ^and ER^- ^PR^- ^receptor subtypes [19]. Our effect estimate for alcohol consumption was higher than those reported previously, possibly because our women drinkers consumed greater amounts of alcohol. Experimental [38] and cross-sectional [39] data have shown that alcohol consumption may result in an increase in blood estrogen levels among premenopausal women. Our results are consistent with the hypothesis that alcohol exerts its effect on the premenopausal breast via estrogen.

Our finding that late age at menarche is associated with a reduced risk of both ER^+^PR^+ ^and ER^- ^PR^- ^breast cancer is consistent with two [19,20] of four previous case–control studies conducted among women under 45 years [19,20] or premenopausal women [12,22]. The magnitude of the risk reduction in the two previous studies ranged from 20 to 40% for women with the oldest versus the youngest ages at menarche [19,20]. The protective effects of breastfeeding on both receptor-positive and receptor-negative cancers in our study were also observed in previous studies among young [19,21] or premenopausal [12] parous women, although the associations were not statistically significant in two of these studies [12,19]. The magnitude of the protective effects of breastfeeding was larger in our study than was reported from previous studies. Three [12,20,22] of four previous studies also found no association between OC use and ER^+^PR^+ ^or ER^-^PR^- ^breast cancer among young [19,20] or premenopausal women [12,22]; the only inconsistent study found a marginally statistically significant increased risk of ER^- ^PR^- ^cancer among women who had ever used oral contraceptives [19].

It has been argued [10,12] that if certain hormone-related factors predominantly act through estrogen and progesterone mediated by their respective receptors, then these hormone-related factors will be associated with hormone receptor-positive, but not with receptor-negative, breast cancer. Our findings that increasing number of full-term pregnancies is associated with lower risk of ER^+^PR^+ ^cancer, whereas increasing recent average weekly alcohol consumption is associated with greater risk, and that ER^- ^PR^- ^cancers are not affected by these risk factors, support the hypothesis that these factors act predominantly through this type of hormonal mechanism.

In contrast, it could be hypothesized that hormonal factors should affect receptor-positive and receptor-negative breast cancer similarly. It has been hypothesized that an ER^- ^stem cell population gives rise to ER^+ ^progenitor cells [40], which will proliferate when exposed to estrogen, but can also send paracrine signals that will cause neighboring populations of ER^- ^cells to proliferate. Thus, late age at menarche and breastfeeding may still act through hormonal mechanisms that involve ER and PR, but our results suggest that the exact mechanism differs from that involved in the effect of parity and alcohol consumption.

One strength of our study is the large number of case patients included. Our analysis included more young breast cancer patients than six of the eight previously published studies with results for ER/PR status among women under the age of 50 years [19-21,36] or among premenopausal women [12,22,33,37]. For the only two studies with a larger sample size, one focused exclusively on alcohol consumption [36] and the other focused on reproductive factors including parity, age at first full-term pregnancy, and breastfeeding [21].

Several limitations of this study must also be considered. The number of control subjects in this study was relatively small. This could explain why we detected a similar magnitude of effect for late first full-term pregnancy on ER^+^PR^+ ^cancer as the Women's CARE Study [21], but in our study it did not reach statistical significance because of our limited statistical power. This could also explain why our case–case analyses suggested that the effect of late age at first full-term pregnancy and BMI differed significantly between the ER^+^PR^+ ^and the ER^- ^PR^- ^case patients, whereas we did not detect any statistically significant associations when comparing each of the subtypes with the control subjects.

Because of our decision not to retain the case–control match during data analyses, we used an unconditional instead of a conditional logistic regression approach, which could have biased our relative risk estimates toward the null value, as described by Rothman and Greenland [29]. However, in comparison with the data from the Women's CARE Study [21], which has so far been the largest population-based case–control study of women aged 35 to 64 years, we found similar results for parity and breastfeeding, whereas the positive association between late first full-term pregnancy and ER^+^PR^+ ^cancer was statistically significant in the Women's CARE Study but not in ours. In comparison with the prospective data from the Nurses' Health Study [18] for both premenopausal and postmenopausal women, our results for age at menarche and parity were consistent with theirs, but our results for late first full-term pregnancy were not. In addition, our overall findings for all the hormone-related factors we examined are quite similar to those in the literature for young or premenopausal women.

We have no data on the methods and cutoff points for receptor status used by each laboratory because we obtained this information from the CSP, which bases its classifications on information in pathology reports from a variety of laboratories. Although we assume that most laboratories used immunohistochemistry assays and consistent cutoff points, it is possible that some laboratories used different methods or different cutoff points. However, we believe that any such inconsistencies would be unlikely to cause the observed associations and, if anything, that they would bias our relative risk estimates toward the null value.

Another limitation is that in our analyses by receptor subtypes, we excluded 21% of our case patients, because 16% of patients had no ER or PR status information and 5% were borderline positives or undecided for either ER or PR. The percentage of case patients without information from the cancer registry in this study (16%) is similar to that reported by previous studies conducted within the SEER registries [21,28]. It is unclear why some case patients do not have a known ER/PR status. We observed that, in this study, the case patients with known ER/PR status information were somewhat younger, were better educated, gave birth later, and were more likely to breastfeed than those whose ER/PR status had not been determined. These differences between case patients with known ER/PR status and those without ER/PR information from the CSP could have biased our case–control comparison by receptor subtypes and caused us to find an effect of late age at first full-term pregnancy even if none existed, but would tend to underestimate any protective effect of breastfeeding on breast cancer risk. It is unlikely that this bias would be different for ER^+^PR^+ ^and ER^- ^PR^- ^cancer. Furthermore, this would not have influenced our case–control analysis using all case patients combined. Because our results from the case–case and the case–control analysis by receptor subtypes or using all case patients combined were generally consistent, we think it unlikely that these issues caused important bias in this study.

## Conclusion

Our results suggest that the number of full-term pregnancies and recent alcohol consumption affect breast cancer risk in young women predominantly through estrogen and progesterone mediated by their respective receptors. Late age at menarche and breastfeeding may protect against breast cancer through a different hormonal mechanism.

## Abbreviations

BMI = body mass index; CARE = Contraceptive and Reproductive Experiences; CI = confidence interval; COC = combination oral contraceptive; CSP = Cancer Surveillance Program; ER = estrogen receptor; OR = odds ratio; PR = progesterone receptor; SEER = Surveillance, Epidemiology, and End Results.

## Competing interests

The authors declare that they have no competing interests.

## Authors' contributions

HM cleaned the data, performed the data analysis, and drafted the manuscript. LB and RKR participated in the design of the study and revised the manuscript. GU designed the study, supervised the data collection and data analysis, and revised the manuscript. All authors read and approved the final manuscript.

## Figures and Tables

**Table 1:** Demographic and hormone-related risk factors

Variables	Cases by availability of ER/PR information				Controls
		
	Cases with known receptor status	Cases with borderline positive/undecided status	Cases without information	*p *for comparison across the three case groups	
Number of subjects	1,366 (79.2%)	83 (4.8%)	276 (16.0%)		440
Mean age at reference date (SD), years	42.5 (5.4)	42.8 (5.2)	44.0 (4.6)	0.0001^a,b^	42.6 (4.9)
Race					
White	1,206 (88.3%)	77 (92.8%)	238 (86.2%)		405 (92.0%)
African-American	160 (11.7%)	6 (7.2%)	38 (13.8%)	0.26^c^	35 (8.0%)
Education					
High school or lower	262 (19.2%)	19 (22.9%)	76 (27.5%)		60 (13.6%)
Technical school or some college	489 (35.8%)	28 (33.7%)	103 (37.3%)		150 (34.1%)
College graduate	615 (45.0%)	36 (43.4%)	97 (35.2%)	0.01^c,d^	230 (52.3%)
First-degree breast cancer family history					
No	1,091 (79.9%)	63 (75.9%)	224 (81.1%)		388 (88.2%)
Yes	229 (16.7%)	19 (22.9%)	46 (16.7%)		41 (9.3%)
Unknown^e^	46 (3.4%)	1 (1.2%)	6 (2.2%)	0.39^c^	11 (2.5%)
Mean age at menarche (SD), years	12.4 (1.5)	12.6 (1.7)	12.4 (1.4)	0.51^a^	12.7 (1.5)
Ever had a full-term (>26-week) pregnancy	66.6%	74.7%	68.5%	0.28^c^	71.6%
Mean number of full-term pregnancies (SD)	2.1 (1.0)	2.1 (1.1)	2.1 (0.9)	0.92^a^	2.2 (1.1)
Mean age at first full-term pregnancy (SD)	26.7 (6.4)	26.2 (6.2)	24.9 (5.8)	0.002^a,b^	27.1 (6.2)
Ever breastfed among parous women	80.4%	77.4%	70.9%	0.01^c,d^	87.9%
Mean duration of breastfeeding (all pregnancies) among those who breastfed (SD), months	14.2 (16.3)	13.7 (13.6)	12.6 (14.6)	0.56^a^	16.6 (17.3)
Mean BMI 1 year before reference date (SD), kg/m^2^	25.8 (6.1)	26.2 (6.4)	26.6 (6.5)	0.12^a^	25.7 (6.4)
Ever used COC	85.9%	84.3%	85.9%	0.92^c^	89.3%
Mean duration of COC use among users (SD), years	6.9 (6.3)	5.8 (6.6)	6.4 (6.4)	0.23^a^	6.7 (6.2)
Alcohol drinking status during reference age					
Never	428 (31.3%)	34 (41.0%)	96 (37.8%)		141 (32.1%)
Former	259 (19.0%)	16 (19.3%)	53 (19.2%)		90 (20.5%)
Current	679 (49.7%)	33 (39.7%)	127 (46.0%)	0.30^c^	209 (47.5%)
Alcohol drinking status in recent 5-year period					
No	428 (31.3%)	34 (41.0%)	96 (34.8%)		141 (32.1%)
Yes	759 (55.6%)	40 (48.2%)	137 (49.6%)		228 (51.8%)
Yes, but not in the five year period	179 (13.1%)	9 (10.8%)	43 (15.6%)	0.17^c^	71 (16.1%)
Mean number of alcoholic drinks per week in recent 5 years for drinkers (SD)	6.6 (20.1)	4.9 (6.1)	6.0 (8.4)	0.80^a^	4.6 (9.1)

Results are for 1,725 case patients and 440 control subjects. BMI, body mass index; COC, combined oral contraceptive; ER/PR, estrogen receptor and progesterone receptor; SD, standard deviation. ^a^*F *test. ^b^Bonferroni *t *tests for pairwise comparisons: *p *values for cases with complete ER/PR information versus cases without ER/PR information ≤ 0.001, and *p *values for the other comparisons ≥ 0.20. ^c^Pearson χ^2 ^test. ^d^Pearson χ^2 ^tests for pairwise comparisons: *p *values for cases with complete ER/PR information versus cases without ER/PR information ≤ 0.004, and *p *values for the other comparisons ≥ 0.32. ^e^Unknown category was excluded from Pearson χ^2 ^tests.

**Table 2:** Adjusted ORs for breast cancer associated with hormone-related risk factors by ER/PR status

Variables	No. controls	All cases versus controls		ER^+^PR^+ ^cases versus controls		ER^- ^PR^- ^cases versus controls		ER^+^PR^+ ^cases versus ER^- ^PR^- ^cases
		
		Number of cases	OR (95% CI)	Number of cases	OR (95% CI)	Number of cases	OR (95% CI)	OR (95% CI)
Age at menarche, years								
≤ 11	90	400	1.00	188	1.00	92	1.00	1.00
12	109	532	1.13 (0.83–1.55)	278	1.24 (0.88–1.75)	119	1.18 (0.79–1.77)	1.04 (0.74–1.47)
13	121	475	0.88 (0.65–1.21)	231	0.87 (0.62–1.24)	110	0.94 (0.62–1.41)	0.93 (0.65–1.33)
≥ 14	120	318	0.61 (0.44–0.85)	157	0.60 (0.42–0.86)	64	0.59 (0.38–0.92)	1.02 (0.67–1.53)
Trend *p *value			0.0007		0.0006		0.008	0.85
Number of full-term pregnancies^a^								
None	125	564	1.00	301	1.00	113	1.00	1.00
1	85	347	0.91 (0.66–1.25)	157	0.77 (0.55–1.10)	82	1.06 (0.70–1.62)	0.70 (0.49–1.02)
2	139	492	0.81 (0.61–1.07)	251	0.77 (0.57–1.06)	106	0.95 (0.64–1.39)	0.84 (0.60–1.18)
3	56	224	0.81 (0.56–1.18)	100	0.69 (0.45–1.04)	54	1.06 (0.65–1.73)	0.68 (0.44–1.05)
≥ 4	35	98	0.49 (0.31–0.78)	45	0.47 (0.28–0.80)	30	0.72 (0.39–1.33)	0.65 (0.37–1.15)
Trend *p *value			0.008		0.005		0.50	0.09
Age at first full-term pregnancy for parous women, years^a,b^								
<22	68	316	1.00	126	1.00	96	1.00	1.00
22–27	89	333	1.07 (0.72–1.58)	149	1.14 (0.73–1.78)	81	0.92 (0.56–1.50)	1.25 (0.81–1.94)
28–31	78	265	1.03 (0.66–1.60)	134	1.17 (0.71–1.93)	57	0.82 (0.46–1.45)	1.42 (0.85–2.37)
≥ 32	80	247	0.92 (0.57–1.48)	144	1.23 (0.72–2.10)	38	0.56 (0.30–1.07)	2.22 (1.24–3.98)
Trend *p *value			0.65		0.49		0.08	0.009
Duration of breastfeeding for parous women, months^a,b^								
0	38	247	1.00	102	1.00	63	1.00	1.00
<1	23	144	0.99 (0.56–1.77)	65	1.01 (0.53–1.90)	38	1.19 (0.59–2.39)	0.82 (0.47–1.44)
1–6	80	273	0.58 (0.37–0.91)	131	0.57 (0.34–0.94)	68	0.72 (0.41–1.27)	0.77 (0.47–1.26)
7–23	110	322	0.52 (0.33–0.82)	165	0.52 (0.31–0.87)	63	0.55 (0.31–0.98)	0.97 (0.59–1.61)
≥ 24	64	175	0.51 (0.30–0.86)	90	0.49 (0.27–0.87)	40	0.62 (0.32–1.21)	0.77 (0.42–1.39)
Trend *p *value			0.001		0.002		0.03	0.63
BMI 1 year before reference date, kg/m^2^								
<25	257	939	1.00	495	1.00	183	1.00	1.00
25–29	95	425	1.18 (0.89–1.55)	209	1.11 (0.82–1.50)	101	1.41 (0.99–2.02)	0.81 (0.59–1.10)
30–34	51	221	1.01 (0.70–1.44)	94	0.88 (0.59–1.30)	61	1.43 (0.91–2.23)	0.60 (0.40–0.89)
≥ 35	37	140	0.88 (0.58–1.34)	56	0.69 (0.43–1.11)	40	1.18 (0.70–2.01)	0.61 (0.38–0.99)
Trend *p *value			0.82		0.20		0.16	0.005
Duration of COC use, years								
Never	47	244	1.00	128	1.00	47	1.00	1.00
<1	73	295	0.78 (0.51–1.18)	133	0.70 (0.44–1.12)	59	0.76 (0.43–1.33)	0.93 (0.57–1.52)
1–4	108	429	0.80 (0.54–1.19)	227	0.81 (0.53–1.25)	90	0.87 (0.52–1.46)	0.92 (0.59–1.43)
5–9	115	362	0.62 (0.42–0.93)	173	0.57 (0.37–0.88)	90	0.82 (0.49–1.37)	0.71 (0.45–1.12)
≥ 10	97	395	0.84 (0.56–1.26)	193	0.76 (0.49–1.18)	99	1.27 (0.75–2.14)	0.61 (0.39–0.96)
Trend *p *value			0.30		0.16		0.18	0.008
Alcohol drinking status during reference age^c^								
Never	141	558	1.00	251	1.00	138	1.00	1.00
Former	90	328	0.96 (0.71–1.31)	162	1.02 (0.72–1.44)	74	0.88 (0.59–1.33)	1.14 (0.79–1.64)
Current	209	839	1.05 (0.81–1.36)	441	1.17 (0.88–1.55)	173	0.93 (0.67–1.30)	1.25 (0.93–1.68)
Average number of alcoholic drinks per week in recent 5 years								
Never	141	558	1.00	251	1.00	138	1.00	1.00
<3	130	481	1.01 (0.76–1.35)	248	1.11 (0.81–1.53)	101	0.89 (0.61–1.30)	1.22 (0.87–1.71)
3–5	49	171	0.93 (0.63–1.37)	90	1.01 (0.66–1.54)	34	0.76 (0.45–1.28)	1.31 (0.81–2.11)
6–11	32	157	1.16 (0.75–1.81)	82	1.26 (0.78–2.03)	34	1.06 (0.60–1.86)	1.20 (0.74–1.94)
> 12	17	127	1.77 (1.01–3.08)	68	2.10 (1.17–3.79)	27	1.71 (0.87–3.38)	1.24 (0.73–2.09)
Trend *p *value			0.12		0.03		0.42	0.23
Drinking alcohol but not in the recent five years	71	231	0.88 (0.63–1.22)	115	0.92 (0.63–1.34)	51	0.80 (0.51–1.26)	1.14 (0.76–1.73)

ORs are from multivariable unconditional logistic regression models with the adjustment for race, age, education, first-degree breast cancer family history, age at menarche, gravidity, number of full-term pregnancies, BMI 1 year before reference date, COC use, the average number of alcoholic drinks per week in the recent 5 years, a variable combining menopausal status and hormone therapy use except where otherwise noted. BMI, body mass index; COC, combined oral contraceptive; CI, confidence interval; ER, estrogen receptor; OR, odds ratio; PR, progesterone receptor. ^a^Models did not include gravidity. ^b^Age at first full-term pregnancy and duration of breastfeeding mutually adjusted for each other. ^c^Alcohol drinking status during reference age replaced the number of alcoholic drinks per week in the recent 5 years in the models.
